# Unilateral Virginal Breast Hypertrophy: A Report of a Rare Condition With an Unknown Etiology

**DOI:** 10.7759/cureus.71809

**Published:** 2024-10-18

**Authors:** Prajakta P Kirdat Patil, Pratiksha Yadav, Varsha Rangankar, Archana C. Buch

**Affiliations:** 1 Radiodiagnosis, Dr. D. Y. Patil Medical College, Hospital and Research Centre, Dr. D. Y. Patil Vidyapeeth (Deemed to be University), Pune, IND; 2 Interventional Radiology, Dr. D. Y. Patil Medical College, Hospital and Research Centre, Dr. D. Y. Patil Vidyapeeth (Deemed to be University), Pune, IND; 3 Pathology, Dr. D. Y. Patil Medical College, Hospital and Research Centre, Dr. D. Y. Patil Vidyapeeth (Deemed to be University), Pune, IND

**Keywords:** abnormal breast development, benign breast condition, breast disease, breast reduction surgery (brs), giant fibroadenoma, gigantomastia, macromastia, unilateral virginal hypertrophy

## Abstract

Unilateral virginal hypertrophy of the breast is a rare condition with an unknown etiology, though it is thought to result from hypersensitivity of the end organs to normal levels of gonadal hormones. We present the case of a 17-year-old girl who initially showed gradual growth after thelarche; however, in the last three to four months, there was a rapid increase in size, leading to psychological and physical problems that also impacted her social life. A clinical examination revealed that her left breast was markedly enlarged compared to the right, and she exhibited a drooping posture. Radiological investigations and a true-cut biopsy were performed to detect any underlying pathology, which revealed normocellular stroma consistent with virginal breast hypertrophy.

## Introduction

Virginal hypertrophy of the breast or gigantomastia is a rare, benign condition of unknown etiology that typically occurs in young pubescent girls. Durston first described it in 1669 [1]. Breast hypertrophy usually occurs in puberty or following pregnancy. It is defined as massive enlargement of the breast tissue requiring a reduction of 1800 g per side of breast volume [2]. Most of these cases are sporadic, and glandular tissue is abnormally sensitive to the pubertal hormones [3]. Although it usually affects both breasts, cases of unilateral growth have also been observed. This condition differs from unilateral breast enlargement caused by underlying pathologies such as fibroadenoma, cystosarcoma phyllodes, or breast cancer, which present with palpable lumps. The most common clinical presentation is a rapid increase in breast size, which further stretches the overlying skin and may cause areolar abnormality, ulceration, and pain. Unilateral breast enlargement can lead to various problems, including abnormal posture, breast discomfort, neck pain, and abnormal spinal curvature, as well as significant impacts on the patient's psychological and social life [3]. We are presenting a case of unilateral virginal hypertrophy of the breast in a pubertal girl.

## Case presentation

**Figure 1:**
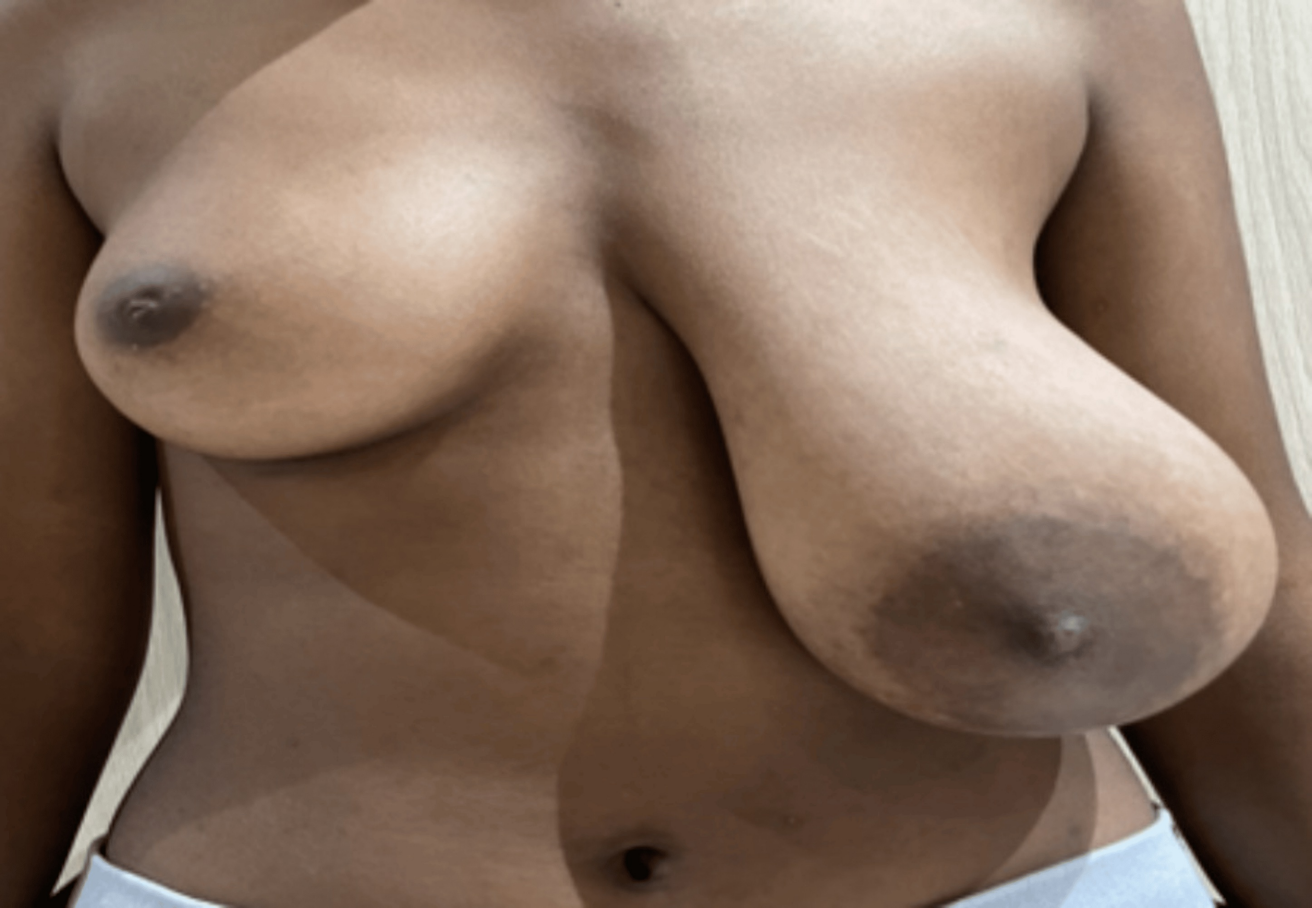
Clinical picture of the patient demonstrating the asymmetrical enlargement of her left breast.

A young, 17-year-old girl presented with complaints of a marked enlargement of her left breast, and she suspected a lump in her left breast that had been increasing in size for the past three months. She had her menarche and thelarche at the age of 11, and her menstrual cycles had been regular. There was no significant genetic, medical, or family history. On clinical examination, there was a marked enlargement of the left breast compared to the contralateral breast (Figure 1). There was enlargement of the areola as well on the left side. Both breasts were soft with no palpable lumps, pain, or tenderness. No abnormal skin changes were noted.

**Figure 2:**
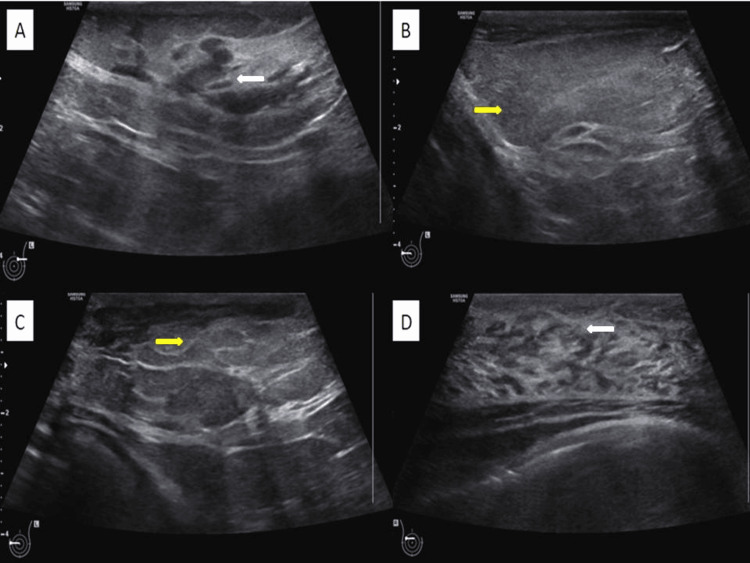
A) High-resolution ultrasound of the left breast upper outer quadrant showing an asymmetrical distribution of fibro-glandular tissue (white arrow). B) and C) The rest of the left breast was showing fatty breast tissue (yellow arrow). D) Normal fibro-glandular tissue seen in the contralateral breast (white arrow).

She had no history of nipple discharge or constitutional symptoms. Her parents and the girl were concerned about the asymmetry of the breast size and its impact on social life associated with the possibility of a cancerous lump. She was therefore recommended a breast ultrasound scan and Tru-cut biopsy. The ultrasound (USG) examination of the breast revealed a significantly enlarged left breast. High-resolution USG of the left breast showed an asymmetrical distribution of fibro-glandular tissue, which was seen more in the upper outer quadrant (Figure 2A). The rest of the left breast showed predominantly fatty breast tissue ( Figure 2B, 2C). The right breast appeared normal according to her age (Tanner stage 5) and physical constitution. The USG examination of the right breast revealed normal fibro-glandular tissue (Figure 2D). No cystic or solid mass was noted in either breast. Both axillae appeared normal.

**Figure 3:**
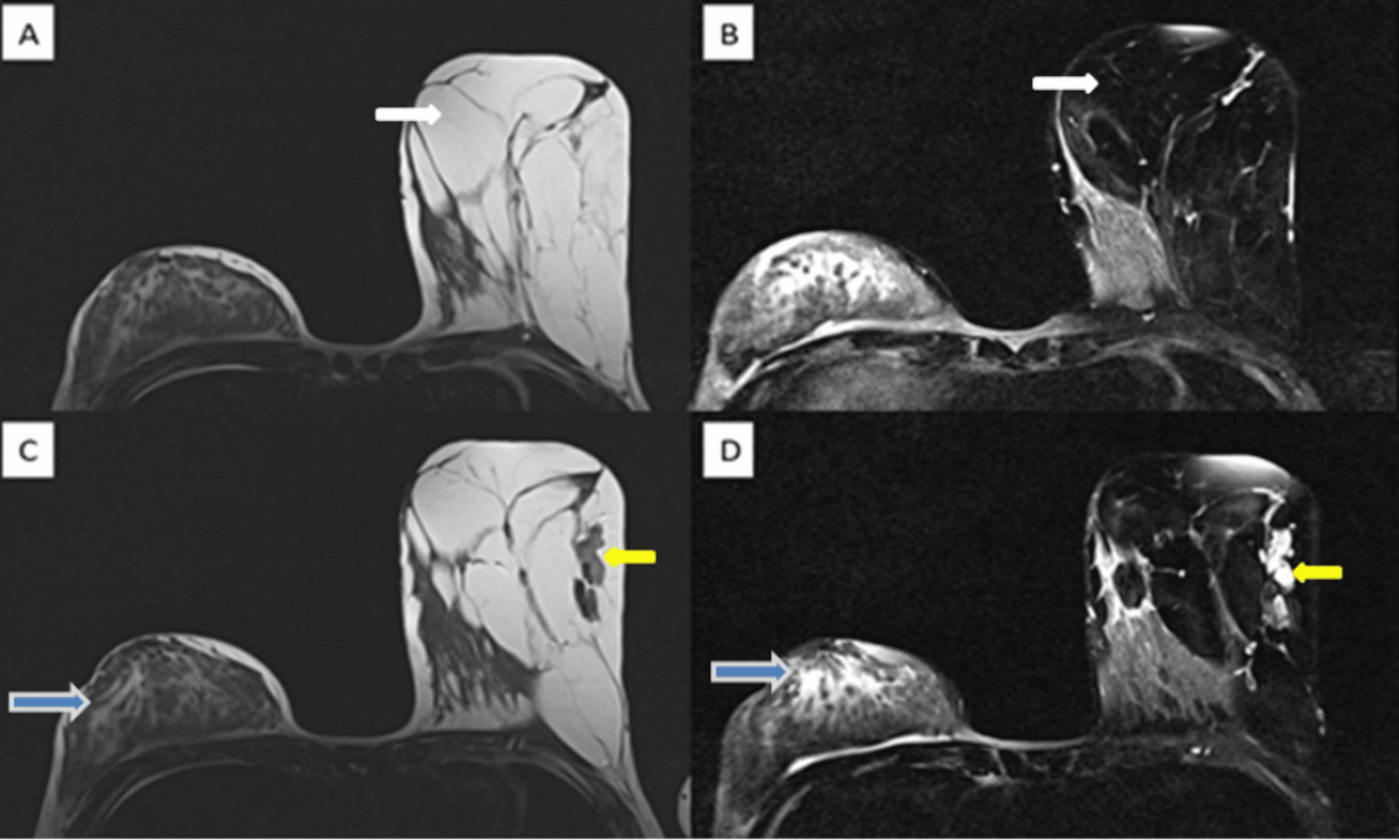
A) Axial T2WI MRI of breast showing marked enlargement of the left breast. B) Axial T2FS MRI showing the suppression of the fatty tissue, which is predominantly occupying the breast (white arrow). C) and D) Axial T2WI MRI and Axial T2FS MRI images showing an asymmetrical distribution of fibro-glandular tissue (yellow arrow). D) Normal fibro-glandular tissue seen in the contralateral breast (blue arrow).

A non-contrast magnetic resonance imaging (MRI) scan was performed for screening and to confirm the USG findings. The MRI revealed significant enlargement of the left breast compared to the right, with predominantly fatty and fibrous tissue throughout. Glandular tissue was primarily scattered in the upper outer quadrant (Figure 3). No cystic or solid masses were observed, and there was no ductal dilatation. The fibrous-glandular pattern in the contralateral right breast was consistent with the patient's age.

**Figure 4:**
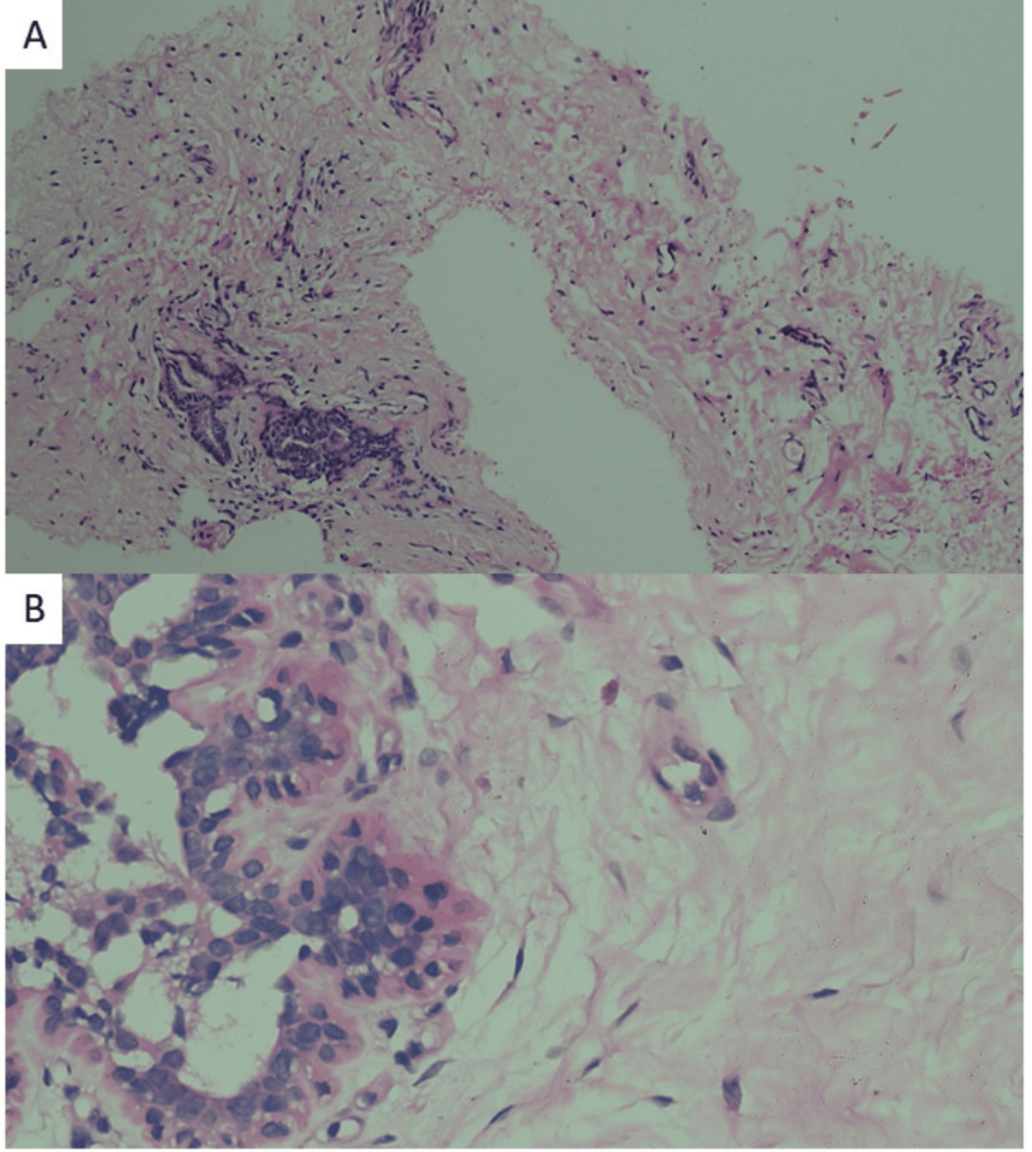
A) Photomicrograph showing normal breast tissue. B) Normal acini lined by cuboidal cells and outer myoepithelial cells.

For further evaluation and pathological diagnosis, a USG-guided needle biopsy of the left breast was performed under local anesthesia. Three gray-white linear tissue cores, measuring 0.5 cm to 1.2 cm, were obtained and placed in 10% neutral buffered formalin (Figure 4). The tissue was routinely processed for histopathology, with slides prepared and stained using hematoxylin and eosin (H&E). The microscopic examination revealed fibrous and fibrocollagenous tissue with a few breast acini lined by cuboidal cells and an outer myoepithelial cell layer. The stroma was normocellular and unremarkable.

After multiple investigations, it was well established that the patient did not have any underlying pathology causing enlargement of the breast. Traditionally, treatment options include breast reduction, mastectomy, reconstruction, hormone therapy, or a combination of these. In this case, the patient was financially unable to afford surgery. She also expressed hesitation about undergoing a surgical procedure; hence, surgery was not performed.

## Discussion

Virginal mammary hypertrophy, also known as juvenile mammary hypertrophy or juvenile gigantomastia, is characterized by the rapid enlargement of one or both breasts during adolescence. Very few cases of unilateral breast enlargement have been reported [4-6]. The development of virginal breast hypertrophy (VBH) is believed to be influenced by hormonal imbalances, genetic factors, and local factors [2]. It typically appears in early adolescence, coinciding with hormonal changes. The excessive growth of the breast is influenced by hormones such as estrogen, progesterone, prolactin, and growth hormones during this rapid growth phase [7]. Excessive breast enlargement can result in physical discomfort, back pain, and poor posture. Additionally, individuals affected by this condition may experience emotional distress, body image issues, and diminished self-esteem [7,8]. 

Imaging studies play a limited role in diagnosing juvenile breast hypertrophy but are essential for ruling out tumors. Mammography can present challenges in interpretation for young women due to dense breast tissue [9]. Breast USG provides valuable information for differential diagnosis and is particularly useful when discrete masses are present and their nature is uncertain. However, excessive breast enlargement can sometimes make USG challenging. Despite this, USG can guide percutaneous biopsy of suspected lesions. MRI is more effective in defining breast architecture and identifying pathological lesions [10]. 

It is crucial to rule out other causes of unilateral breast enlargement, such as large fibroadenoma, cystosarcoma phyllodes, lipoma, or breast hamartoma. Additionally, a distinction must be made between gravid and virginal gigantomastia. Imaging studies are important for treatment planning and preventing long-term complications. Traditionally, treatment options include breast reduction, mastectomy, reconstruction, hormone therapy, or a combination of these. The use of hormonal agents such as tamoxifen in patients who have not yet reached sexual and physical maturity carries risks. These potential adverse effects include endometrial hyperplasia, endometrial cancer, an increased risk of venous thrombosis, and the possibility of triggering depression [10-12]. Surgery is considered the primary treatment approach. Subcutaneous mastectomy involves the complete removal of breast tissue and is least likely to lead to recurrence, but it may deform the breast shape. Reduction mammoplasty offers better aesthetic outcomes but has a higher likelihood of recurrence, which should be communicated to the patient.

## Conclusions

VBH is a relatively rare but significant female health problem, particularly in developing countries where poverty and delayed medical intervention are common problems. The considerable cosmetic impact and associated self-esteem issues are exacerbated in resource-poor settings where delayed medical treatment and inadequate follow-up care are common. Proper assessment of VBH in conjunction with other benign breast conditions is critical to rule out malignancy and ensure timely and appropriate treatment to minimize complications. A comprehensive, multidisciplinary approach is essential for adequate management.
